# Biomarker Expression in Multifocal Vulvar High-Grade Squamous Intraepithelial Lesions

**DOI:** 10.3390/cancers13225646

**Published:** 2021-11-11

**Authors:** Nikki B. Thuijs, Willemijn A. M. Schonck, Linde L. J. Klaver, Guus Fons, Marc van Beurden, Renske D. M. Steenbergen, Maaike C. G. Bleeker

**Affiliations:** 1 Department of Pathology, Cancer Center Amsterdam, Vrije Universiteit Amsterdam, Amsterdam UMC, De Boelelaan 1117, 1081 HV Amsterdam, The Netherlands; n.thuijs@amsterdamumc.nl (N.B.T.); w.schonck@nki.nl (W.A.M.S.); l.klaver@amsterdamumc.nl (L.L.J.K.); r.steenbergen@amsterdamumc.nl (R.D.M.S.); 2 Department of Gynecology, Universiteit van Amsterdam, Amsterdam UMC, Meibergdreef 9, 1105 AZ Amsterdam, The Netherlands; g.fons@amsterdamumc.nl; 3 Department of Gynecology, Antoni van Leeuwenhoek Hospital, Plesmanlaan 121, 1066 CX Amsterdam, The Netherlands; m.v.beurden@nki.nl

**Keywords:** vulvar intraepithelial neoplasia, HPV, biomarker, methylation

## Abstract

**Simple Summary:**

In this exploratory study, we aimed to compare biomarker profiles in patients with multiple high-risk human papillomavirus (HPV)-associated vulvar precursor lesions, which is called multifocal high-grade squamous intraepithelial lesion (HSIL). The HPV-positive HSILs were tested for HPV genotype, expression of two immunohistochemical markers p16^INK4a^ and Ki-67, and DNA methylation of six genes. Generally, the biomarkers showed similar expression between lesions. Occasionally, marked differences were observed, indicating that not all multifocal lesions are the same. Our study contributes to a better understanding of the value of potential diagnostic, prognostic, and predictive biomarkers in patients with vulvar multifocal HSIL. Validation in larger cohorts will be important.

**Abstract:**

In patients with high-grade squamous intraepithelial lesion (HSIL) of the vulva, the presence of multiple lesions, called multifocal HSIL, is common. The aim of this exploratory study was to investigate biomarker expression profiles in multifocal HSIL. In total, 27 lesions from 12 patients with high-risk human papillomavirus (HPV)-positive multifocal HSIL were tested for HPV genotype, expression of p16^INK4a^ and Ki-67, and DNA methylation of six genes. HPV16 was found most commonly in 21 (77.8%) HSILs. In two (16.4%) patients, HPV genotype differed between the lesions. All lesions demonstrated diffuse p16^INK4a^ staining, of which three (11.1%) were combined with patchy staining. One patient (8.3%) demonstrated markedly different DNA methylation levels between lesions. Generally, heterogeneity in methylation profiles was observed between different patients, even when other biomarkers showed similar expression. In conclusion, this study is the first to demonstrate heterogeneity of individual lesions in patients with multifocal HSIL. The studied biomarkers have the potential to refine prognostic and predictive diagnostics. Future prospective, longitudinal studies are needed to further explore the potential of a biomarker profile for management of patients with multifocal HSIL.

## 1. Introduction

High-grade vulvar intraepithelial neoplasia (VIN) is the precursor of vulvar squamous cell carcinoma (VSCC). High-grade VIN is categorized into vulvar high-grade squamous intraepithelial lesion (HSIL), which is human papillomavirus (HPV)-associated, and differentiated VIN (dVIN), which is HPV-independent and associated with lichen sclerosus (LS) [1,2,3]. HSIL, also known as usual type VIN (uVIN), is the most common type of VIN and occurs mainly in patients who smoke from age 35 to 50 years [4]. The presence of multiple HSILs, a frequent finding at clinical examination, is called multifocal HSIL [5,6,7]. To confirm the clinical diagnosis and to exclude underlying invasive disease, multiple biopsies or a so-called vulvar mapping is frequently performed in patients with multifocal HSIL. Treatment options for vulvar HSIL vary from topical imiquimod to surgery, the latter often leading to somatic and psychosexual morbidity [8,9].

HPV infection is found in more than 80% of HSILs, and HPV genotypes 16 and 18 are the most commonly identified [10,11]. Immunostaining of p16^INK4a^ is often used as a surrogate marker of HPV-dependent high-grade intraepithelial lesions [12]. In addition to a persistent, transforming infection with high-risk HPV being a necessary cause for the development of HPV-associated vulvar cancer, genetic and epigenetic alterations in host cell genes are crucial for progression of precancerous lesions to invasive cancer [13,14,15]. DNA methylation is an epigenetic process that regulates gene expression and plays an important role in carcinogenesiss [13]. Gene silencing leads to loss of the tumor suppressive function, thereby contributing to cancer development [13]. In recent years, DNA methylation of specific genes has shown to be a promising biomarker in the identification of anogenital lesions, including vulvar neoplasia [13,15,16,17,18]. It has been demonstrated that methylation levels of 12 genes significantly increase from healthy vulvar tissue toward vulvar cancer [15]. Of these genes, *GHSR, SST, ZIC1, ASCL1, LHX8*, and *ZNF582* were most promising for HSIL and VSCC detection [15].

The expression of biomarkers in multifocal HSIL has never been studied before; however, this information may have predictive value with regard to the clinical course of individual lesions. Therefore, the aim of this exploratory study was to compare histopathological and molecular characteristics amongst individual lesions of patients with multifocal HSIL, i.e., HPV genotyping, immunohistochemical staining patterns of p16^INK4a^ and Ki-67, and methylation profiles of six genes, *GHSR, SST, ZIC1, ASCL1, LHX8*, and *ZNF582*.

## 2. Materials and Methods

### 2.1. Patients and Samples

This exploratory study included 12 patients with high-risk HPV positive multifocal vulvar HSIL, diagnosed between 1991 and 2005. Only patients with incident HSIL, before treatment interference, were included. In total, 27 lesions, varying from 2–4 lesions per patient, were examined. Multifocal lesions were defined as multiple HSILs separated by unaffected vulvar skin. Excluded were confluent areas of HSIL or when multifocal HSIL could not be determined with certainty. Excluded were also patients with LS and patients with concurrent or prior VSCC. Patients were identified from an historical cohort of patients with vulvar diseases, which has previously been described in detail [4,19]. Further criteria for selection of the 12 patients were a diagnosis of multifocal vulvar HSIL at Amsterdam UMC, location VUmc or location AMC, according to the definition as described above, and availability of tissue samples at the pathology archives of Amsterdam UMC, location VUmc and AMC. The selected biopsies and excisional biopsies of the patients with multifocal HSIL were either diagnostic or excisional/therapeutic. Baseline and follow-up data were extracted from a pseudonymized clinical database using Castor EDC. Patient identity was protected by study-specific unique patient numbers. Tissues were anonymously processed for the purpose of this study. Accordingly, no further patient approval was needed. The local Medical Ethics Committee of Amsterdam UMC, location VUmc, confirmed that the Medical Research Involving Human Subjects Act did not apply to this study and approved the study under reference number 2017.561.

### 2.2. Processing of Tissue Blocks

For contamination-free DNA isolation, whole-tissue sections of formalin-fixed, paraffin embedded (FFPE) tissue blocks were sectioned using the sandwich method. The first and last sections (3 μm) were used for hematoxylin–eosin (H&E) staining to ensure the presence of the lesion. In-between sections were collected in sterile PCR tubes for DNA isolation (10 μm) and for immunostaining (3 μm). Precautions were taken to avoid cross-contamination as described before [20].

### 2.3. Histopathology and Immunohistochemistry of p16^INK4a^ and Ki-67

All H&E and immunohistochemically stained slides were scored by a gynecopathologist (M.C.G.B.) and a senior resident in pathology (N.B.T.).

The Optiview detection kit with the automated 100 BenchMark ULTRA IHC/ISH system (Roche) was used to perform immunostaining of both p16^INK4a^ and Ki-67. Mouse monoclonal antibodies against the p16^INK4a^ antigen (clone E6H4; Roche, Basel, Switzerland) were used for immunostaining of p16^INK4a^. Immunostaining of Ki-67 was performed with mouse monoclonal antibodies against the Ki-67 antigen (clone Ki-67; Dako, Glostrup, Denmark).

A negative or patchy staining pattern of p16^INK4a^ was scored as 0, whereas a diffuse (or block) p16^INK4a^ staining pattern up to the lower third of the epithelium was scored as 1, extending above the lower third of the epithelium was scored as 2, or extending more than two-thirds of epithelium was scored as 3. When a diffuse staining pattern for p16^INK4a^ was present, we also scored whether this pattern was completely diffuse or combined with a negative or patchy staining pattern. Ki-67 expression was scored as not increased (score 0), increased in the lower third (score 1), increased in the lower two-thirds (score 2), or increased in more than two-thirds (score 3) of the epithelium.

### 2.4. DNA Isolation

The in-between sections were used for DNA isolation using the QIAamp DNA FFPE tissue kit (Qiagen, Hilden, Germany) according to the manufacturer’s instructions. DNA was eluted with the easyMAG 3 elution buffer (bioMérieux, Boxtel, The Netherlands). DNA concentrations were measured using Qubit (Thermo Fisher Scientific Inc., Qiagen).

### 2.5. DNA Methylation Analysis

For methylation analysis, isolated DNA was bisulfite-converted using the EZ-DNA Methylation kit (Zymo Research, Orange, CA, USA) [21]. Methylation analysis was performed using EpiTect MethyLight Master Mix (Qiagen, Hilden, Germany), together with fluorescent dry-labelled probes, 50 ng of bisulfite-converted DNA, and 100–300 nM of each primer [22]. Six methylation markers, *GHSR, SST, ZIC1, ASCL1, LHX8*, and *ZNF582*, and the reference gene, β-actin (*ACTB*), were tested by quantitative methylation-specific PCR (qMSP) assays as described previously [22,23]. Samples with a quantification cycle threshold (Ct) of *ACTB* ≤32 indicated sufficient DNA and adequate bisulfite conversion [23]. No invalid test results were obtained. ΔCt ratios were computed using the comparative Ct method, normalizing target Ct values to *ACTB* [24]. Additionally, DNA methylation levels for all genes were categorized into quartiles: ≤25th percentile, 25th–50th percentile, 50th–75th percentile, and >75th percentile.

### 2.6. Human Papillomavirus (HPV) Testing and Genotyping

The QIAscreen^®^ HPV PCR Test (QIAgen, Hilden, Germany) was used to perform high-risk HPV DNA-testing, according to the manufacturer’s instructions. Analysis was directed against the E7 gene of the following high-risk HPV genotypes, i.e., 16, 18, 31, 33, 35, 39, 45, 51, 52, 56, 58, 59, 66, 67, and 68, with partial genotype information (HPV16 and 18) [25]. β-Globin was used for internal quality control.

## 3. Results

### 3.1. Baseline Characteristics

Baseline and follow-up characteristics of the study population are shown in Table 1. Median age at diagnosis of multifocal HSIL was 40 years (range 24–58). In total, 27 lesions of 12 patients were analyzed, varying from 2–4 lesions per patient. Aspects of lesions, including shape, color, and thickness, could be retrieved from the records of seven patients. Topographic sites included the labia minora (*n* = 7), labia majora (*n* = 7), perineum (*n* = 3), commissura posterior (*n* = 2), perianal region (*n* = 1), and clitoris (*n* = 1). Eight patients (66.7%) patients had other HPV-related anogenital conditions, i.e., multicentric disease (squamous intraepithelial lesions of cervix, vagina, or anus) and/or anogenital condylomata acuminata. Two patients (16.7%) were immunocompromised, one by human immunodeficiency virus (HIV) and one by systemic lupus erythematosus (SLE). None of the patients had vulvar LS. Of all patients, eight (75%) had diagnostic biopsies, and four (25%) underwent therapeutic excisional biopsies at baseline. Primary treatment was local excision or skinning vulvectomy in the majority of cases (10/12, 83.3%). Median follow-up time was 21.8 years (range 14.8–26.5). Two patients were cured after primary treatment, whereas the remaining patients suffered from HSIL up to 20.2 years. In total, 4/12 patients (25%) progressed toward vulvar cancer, between 5.9 and 11.4 years after baseline HSIL.

### 3.2. Biomarker Expression

**Figure 1:**
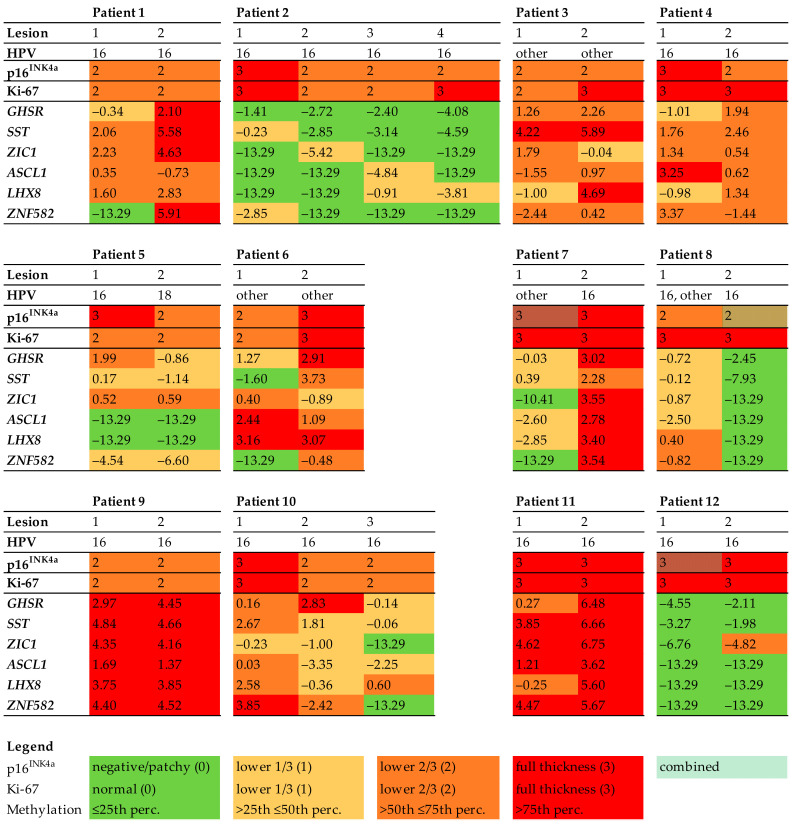
Histopathology and biomarker profiles in 12 patients with multifocal HSIL. Immunohistochemical scores for p16^INK4a^ (0–3) and Ki-67 (0–3), high-risk HPV genotypes, and log_2_ transformed methylation levels of all HSILs. DNA methylation levels for all genes (*GHSR, SST, ZIC1, ASCL1, LHX8,* and *ZNF582*) were categorized into quartiles. Each column represents one HSIL. The colours refer to the biomarker expression, as indicated in the legend. Abbreviations: HPV = human papillomavirus.

**Figure 2:**
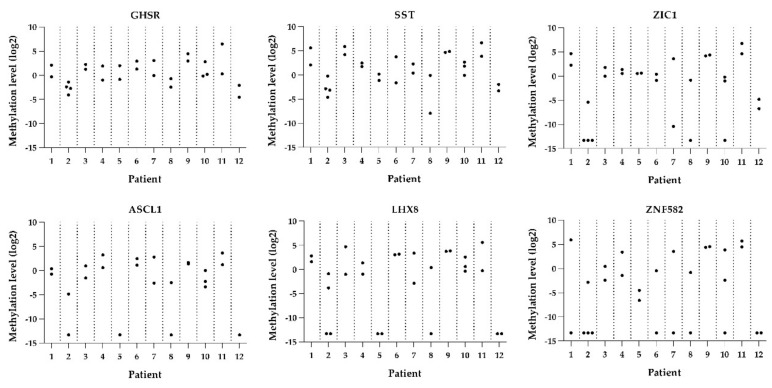
Methylation levels per lesion per patient for all six genes (*GHSR, SST, ZIC1, ASCL1, LHX8,* and *ZNF582)*.

The histopathological and biomarker characteristics of all 27 HSILs are shown in Figure 1 and Figure 2.

Genotyping showed that HPV16 was most common, found in 21 HSILs (77.8%). One of the 27 lesions was HPV18-positive (3.7%). Two patients had a different HPV genotype in each HSIL (16.7%, patients 5 and 7). One patient had multiple HPV genotypes in one HSIL (patient 8, lesion 1).

**Figure 3:**
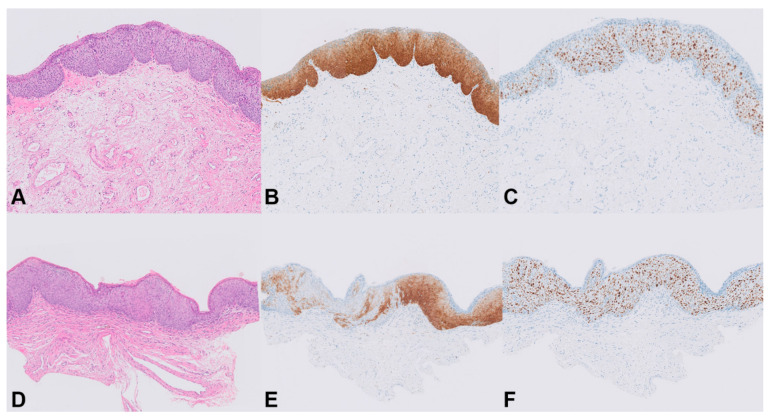
Staining patterns for p16^INK4a^ in HSIL. Patient 8, HSIL 1: (**A**) H&E stain (20×); (**B**) homogeneous p16^INK4a^ staining, score 3 (20×); (**C**) full-thickness immunostaining of Ki-67, score 3 (20×). Patient 8, HSIL 2: (**D**) H&E stain (20×); (**E**) combined diffuse and patchy p16^INK4a^ staining, score 2 (20×); (**F**) full-thickness immunostaining of Ki-67, score 3 (20×).

All HSILs showed diffuse p16^INK4a^ staining in two-thirds or more of the epithelium (score 2 or 3, respectively). In the majority of HSILs (*n* = 24, 88.9%), a completely diffuse staining pattern for p16^INK4a^ was found, whereas three HSILs (11.1%) showed a combined diffuse and patchy pattern (Figure 3). All HSILs showed increased proliferation activity up to two-thirds or more of the epithelium (score 2 or 3, respectively), measured by Ki-67 expression.

DNA methylation levels between HSILs varied from absent (i.e., log_2_ transformed methylation level of −13.29) to high (methylation level of 6.75). Only one patient (patient 7) showed marked methylation differences between the HSILs, with a difference in DNA methylation of at least two quartiles in most (five out of six) markers. The remaining 11 patients had smaller differences in methylation levels or differences in only a few markers between HSILs. Interestingly, all three HSILs with combined diffuse and patchy p16^INK4a^ staining showed lower methylation levels compared to their counterpart HSIL with only diffuse p16^INK4a^ staining (patients 7, 8, and 12). In both HPV16 and non-HPV16, low and high methylation levels were seen and no statistically significant difference in methylation levels between HPV16 and non-HPV16 HSIL was observed. Overall, DNA methylation levels showed a trend toward increased methylation levels with higher p16^INK4a^ expression. However, given the low numbers of lesions, results were not significant.

The four patients who developed vulvar cancer (patients 7, 10, 11, and 12) were all positive for HPV16 and had persistent HSIL for at least 11.4 years. Three of these four patients (patients 7, 10, and 11) had high methylation levels in at least one baseline HSIL. In one of these patients (patient 7), the HSIL with high methylation level was at the same site as the site of vulvar cancer. In the other two patients, this could not be verified with certainty. One patient (patient 12) had low methylation levels in both baseline HSILs and developed vulvar cancer at a different anatomical site after 11.4 years.

## 4. Discussion

This study is the first to have systematically investigated the biomarker expression in individual vulvar high-grade lesions of patients with multifocal HSIL. For most patients with multifocal HSIL, the biomarkers showed comparable expression profiles between lesions. In one patient, remarkable differences in HPV genotype and DNA methylation levels for five out of six markers were observed, while both HSIL morphology and p16^INK4a^/Ki-67 staining patterns were similar.

It is often not possible to reliably diagnose HSIL on the basis of only the clinical aspect of the lesion. The clinical features of HSIL vary in vulvar topography, size, surface, shape, color, and thickness [26]. Therefore, all clinically suspicious lesions are biopsied to confirm the diagnosis and to exclude invasive disease. This results in relatively high diagnostic costs and increased morbidity of patients. Testing for biomarkers might provide objective prognostic and predictive information, which is valuable for the management of patients with HSIL.

Of the 27 high-risk HPV positive HSILs, 78% had HPV16, 3.7% had HPV18, and 22% had another high-risk type. This distribution of HPV genotype is comparable to the literature [10,11]. Two of 12 patients had a different HPV genotype in each HSIL, indicating that these lesions developed independently. One patient had two high-risk HPV genotypes within the same lesion. According to the literature, more than 90% of VIN lesions are attributable to only one HPV genotype [10]. The presence of two HPV types in one lesion may result from a collision of two independent HSILs, each with a unique HPV type. However, the two HPV types detected in one lesion in the present study differed largely in abundance, with highly abundant HPV16 most likely being the single causative type. The low abundance of HPV-other may be explained by the fact that the patient was immunocompromised by systemic lupus erythematodes [27]. In cervical lesions, the presence of multiple HPV genotypes is thought to be associated with persistent high-risk HPV infections, which is probably related to impaired immunity [28,29,30]. The biological relevance of different and multiple HPV genotypes in vulvar lesions has not been studied and remains to be elucidated. Moreover, no data exist on progression risk in HSIL stratified per HPV genotype. In cervical premalignant lesions, the progression risk is highest for HPV16 [31]. In our study, all four patients who progressed toward vulvar cancer were positive for HPV16. No statistically significant difference in methylation levels between HPV16 and other high-risk HPV types was observed, which is likely due to small study numbers.

Consistent with a diagnosis of high-risk HPV-associated HSIL, all lesions stained diffusely positive for p16^INK4a^ and showed increased Ki-67 expression. P16^INK4a^ is frequently used to optimize grading of HPV-induced anogenital lesions, and diffuse staining is considered a surrogate marker for HPV-associated high-grade anogenital lesions [32,33]. The Lower Anogenital Squamous Terminology (LAST) Project only recommends the use of p16^INK4a^ to differentiate between HSIL and LSIL or mimics of precancer [34]. In our study, three of 27 HSILs had a combined diffuse and patchy staining for p16^INK4a^. While it is not clear whether this reflects the biological behavior of these lesions, it can be speculated that these HSILs have a lower malignant potential. Consistently, the lower malignant potential is supported by the very low or negative methylation levels found in these lesions. However, the vast majority of HSILs demonstrated a uniform diffuse p16^INK4a^ staining pattern, while both high and low methylation levels were seen, indicating heterogeneity of vulvar HSILs. This observation is in agreement with earlier studies showing that morphologically identical vulvar HSILs show substantial molecular heterogeneity with respect to both copy number aberrations (CNA) and DNA methylation, despite similar histopathological classification and p16^INK4a^/Ki-67 staining patterns [14,15,18]. This heterogeneity is also seen in cervical and anal p16^INK4a^-positive HSIL, with a subset of those high-grade anogenital lesions having as high methylation levels as cancer [22].

This study had some limitations. The retrospective study design hindered collection of all patient characteristics, preventing us from linking clinical characteristics to the biomarker expression of lesions. Secondly, the study population was too small to draw firm conclusions or to evaluate biomarker results in a multivariate analysis. Thirdly, since we analyzed a cross-sectional series of multifocal HSILs, we could not prove that multifocal HSILs with high expression levels had a higher risk of persistence or progression to cancer compared to multifocal HSILs with low expression levels. Thus, further research on the role of the selected biomarkers in multifocal HSILs during the longitudinal course of vulvar carcinogenesis is needed.

Our study also had several strengths. It is the first study which systematically describes the expression of multiple biomarkers, including HPV genotyping, immunohistochemistry, and DNA methylation, in patients with multifocal vulvar HSIL. Furthermore, we are the first to show the heterogeneity of vulvar HSILs. The studied biomarkers have great potential to refine prognostic and predictive diagnostics.

## 5. Conclusions

In conclusion, this exploratory study demonstrates that heterogeneity between individual lesions of patients with multifocal HSIL exists, albeit only present in a small proportion of those patients. Patients with HSIL yielding high methylation levels may have an increased risk of developing vulvar cancer. Future prospective studies with long-term follow-up and larger sample sizes are needed to further explore the potential value of a biomarker profile for management of patients with multifocal HSIL.

## Figures and Tables

**Table 1:** Baseline and follow-up characteristics of the study population of 12 patients with multifocal HSIL.

					Baseline					Follow-Up			
Patient	Age (years)	Other Anogenital Conditions	Smoking	Immuno-Compromised	Number	Aspect	Topography	Type of Biopsy	Primary Treatment	Time to Last HSIL Diagnosis (years)	VSCC During Follow-Up	Time to VSCC (years)	Topography VSCC
1	42	AIN3, condylomata acuminata	Unknown	Unknown	2	Not specified	Perineum, lab maj R	Diagnostic	Skinning vulvectomy	*	No	NA	NA
2	24	None	Yes	No	4	Hyperpigmentation, maculopapulous	Lab maj R and L, lab min R and L	Diagnostic	Skinning vulvectomy	0.8	No	NA	NA
3	44	AIN2	Yes	No	2	Leukoplakia	Lab min R and L	Diagnostic	Local excision	18.5	No	NA	NA
4	37	CIN3	Yes	No	2	Not specified	6 and 9 o’clock	Diagnostic	Laser evaporatisation	16.7	No	NA	NA
5	58	AIN3, CIN3	Unknown	No	2	Hyperpigmentation, condylomatous brown	Lab min R and L	Diagnostic	None	0.5	No	NA	NA
6	38	None	Yes	No	2	Not specified	Perineum, lab maj/min R	Therapeutic	Local excision	20.2	No	NA	NA
7	45	CIN3, condylomata acuminata	Yes	No	2	Hyperpigmentation	Commisura posterior, perianal	Therapeutic	Local excision	14.8	Yes	9.2	Perianal
8	31	CIN2, VAIN2, condylomata accuminata	Yes	Yes	2	Not specified	Lab maj/min L, lab min R	Diagnostic	Local excision	*	No	NA	NA
9	44	None	Unknown	Unknown	2	Hypertrophic dystrophic	Lab maj R and L	Diagnostic	Local excision	3.1	No	NA	NA
10	49	CIN3	Yes	No	3	Papillomatous, erosive, varyingly pigmented	Perineum, lab maj/min L, lab min R	Diagnostic	Skinning vulvectomy	19.1	Yes	9.3	Perianal
11	38	Unknown	Unknown	Yes	2	Not specified	Lab min R, commissura posterior	Therapeutic	Local excision	11.4	Yes	5.9	Anterior L
12	28	CIN2, condylomata acuminata	Yes	No	2	Hyperpigmentation, condylomatous brown	Lab maj/min L, clitoris	Therapeutic	Skinning vulvectomy	16.1	Yes	11.4	Posterior L

* cured after primary treatment, Abbreviations: AIN = anal intraepithelial neoplasia, CIN = cervical intraepithelial neoplasia, NA = not applicable, VAIN = vaginal epithelial neoplasia, VSCC = vulvar squamous cell carcinoma, lab maj = labium majus, lab min = labium minus, R = right side, L = left side.

## Data Availability

Data can be made available upon reasonable request.
